# Third-Order Theory for the Bending Analysis of Laminated Thin and Thick Plates Including the Strain Gradient Effect

**DOI:** 10.3390/ma14071771

**Published:** 2021-04-03

**Authors:** Michele Bacciocchi, Angelo Marcello Tarantino

**Affiliations:** 1 Dipartimento di Economia, Scienze e Diritto (DESD), University of San Marino, Via Consiglio dei Sessanta, 47891 Dogana, San Marino; 2 Centro di Ricerca Interdipartimentale Costruzioni e del Territorio (CRICT), Via Vivarelli, 41125 Modena, Italy; angelomarcello.tarantino@unimore.it; 3 Department of Engineering “Enzo Ferrari” (DIEF), University of Modena and Reggio Emilia, Via Vivarelli, 41125 Modena, Italy

**Keywords:** laminated composite materials, nonlocal elasticity, strain gradient, thin and thick plates, static analysis

## Abstract

The aim of the paper is the development of a third-order theory for laminated composite plates that is able to accurately investigate their bending behavior in terms of displacements and stresses. The starting point is given by the corresponding Reddy’s Third-order Shear Deformation Theory (TSDT). This model is then generalized to consider simultaneously the Classical Laminated Plate Theory (CLPT), as well as the First-order Shear Deformation Theory (FSDT). The constitutive laws are modified according to the principles of the nonlocal strain gradient approach. The fundamental equations are solved analytically by means of the Navier methodology taking into account cross-ply and angle-ply lamination schemes. The numerical applications are presented to highlight the nonlocal effects on static behavior.

## 1. Introduction

Higher-order plates theories for laminates have been introduced in the last decades to avoid some issues related to the use of lower-order and simpler approaches, such as the Classical Laminated Plate Theory (CLPT) and First-order Shear Deformation Theory (FSDT) [1,2]. In particular, higher-order equivalent single-layer theories allow to obtain a more accurate interlaminar stress analysis and do not need to introduce the shear correction factor [3,4,5,6]. The displacement field that characterizes the Third-order Shear Deformation Theory (TSDT), for instance, determines a quadratic profile of shear strains and stresses along the thickness [7,8], due to its cubic expansion in the thickness coordinate. Consequently, there is no need for the shear correction factor [9,10,11,12]. The importance of these cubic terms in the analysis of laminates has been recently highlighted in the paper by Petrolo and Carrera [13], in which the best theory diagrams for multilayered structures have been widely discussed.

In general, higher-order approaches have been justified by the use of more and more advanced materials [14,15,16,17] and the need of innovative configurations for the optimal design of structures [18,19]. In particular, their introduction could be essential when these innovative constituents are included in the stacking sequences of multilayered or sandwich structures [20,21,22,23,24,25]. These aspects have been clearly emphasized in the works by Carrera [26,27,28], Carrera and Giunta [29], and Carrera et al. [30]. In these works, accurate and effective higher-order structural models based on a unified formulation have been presented.

The increasing number of applications involving micro- and nanostructures [31,32,33,34] has proven that the size-dependent features of the advanced constituents could have not negligible effects on their mechanical behavior [35,36,37]. These aspects have been highlighted by so-called multiscale analysis [38,39,40,41]. In these circumstances, structural theories based on classical elasticity could turn out to be inadequate to model such innovative mediums [42,43,44,45,46,47]. Nonlocal theories have been developed to overcome these issues, as illustrated in the first papers by Eringen [48,49]. Some other subsequent contributions could be mentioned to this aim. For instance, Luciano and Willis investigated the nonlocal constitutive behavior of an infinite composite laminate [50]. Nanorods made of functionally graded materials were studied by Barretta et al. [51], who included the gradient Eringen model in their framework. The same approach was used for Timoshenko nanobeams in bending [52]. Analogously, the effects of nonlocal elasticity were emphasized in the papers by Apuzzo et al. [53,54] for the investigation of torsional behavior and dynamic response of Bernoulli–Euler nanobeams. More recently, similar remarks and observations have been drawn in [55,56,57]. In general, different nonlocal theoretical frameworks can be developed. Examples of nonlocal approaches that should be mentioned for completeness purposes are the strain and stress gradient theories [58,59,60], the modified couple stress theory [61,62,63,64], and the ones based on micropolar formulations [65,66,67]. A comprehensive literature review concerning nonlocal elasticity can be found in the paper by Zhao et al. [68] for completeness purposes.

The current paper is developed within the strain gradient theoretical framework [69]. According to this approach, the size effects of the materials are modeled by means of a length-scale parameter, which controls the second-order derivatives of the strain components [70]. Consequently, the stresses are functions not only of the strains in an evaluation point, but also depend on the divergence of the gradient of the strains. These aspects have been emphasized in the papers by Aifantis [71] and by Askes and Aifantis [72], where an overview of gradient elasticity formulations in statics and dynamics has been presented. Therefore, higher-order derivatives of displacements are involved in the three-dimensional constitutive laws [73,74,75,76]. These aspects have been also recently emphasized in papers [77,78] where peculiar Finite Element (FE) formulations have been developed to take into account the strain gradient effect. In particular, the use of higher-order Hermite interpolating polynomials for the approximation of both membrane and bending degrees of freedom have been disclosed.

The plate theory that this nonlocal effect is included in is based on the TSDT for laminates [1], in which the various layers that define the stacking sequence have orthotropic features [79,80]. The kinematic model is written to take into account simultaneously lower-order approaches, such as the CLPT and FSDT, for comparison purposes. After the brief introduction of the main topics of the paper (Section 1), the theory is presented by using a matrix compact notation in Section 2. Here, the strong form of the governing equations is obtained, once the definitions of both strains and stress resultants are carried out. The fundamental system is solved analytically by means of the Navier approach as shown in [73]. The algebraic system of equations is written in Section 3, highlighting the contributions related to the strain gradient effect for cross-ply and angle-ply simply supported laminated composite plates. As remarked in [1], the Navier methodology can be efficiently applied to deal with these configurations. Section 4 is focused on the numerical applications. The proposed approach is validated for both classical and nonlocal elasticity through comparison with the results available in the literature, taking into account the three different structural models. Then, the results are extended to emphasize the influence of the nonlocal parameter on the static behavior, which is expressed in terms of displacements and stress components. The through-the-thickness stress profiles are also provided. Finally, the main achievements are drawn in Section 5. Appendix A collects instead the analytical expressions of the terms involved in the algebraic formulation for cross-ply and angle-ply laminates.

## 2. Nonlocal Structural Model

**Figure 1:**
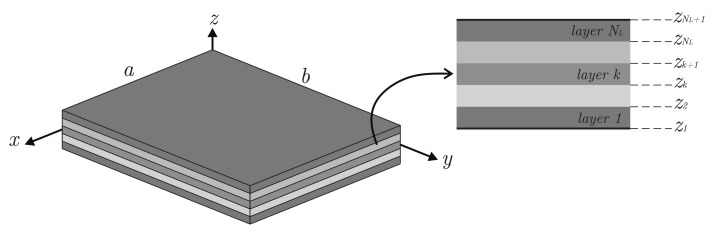
Laminated composite plate: geometry and stacking sequence.

The theoretical framework is developed in this Section for a rectangular plate whose planar size is a×b, considering a Cartesian reference system xyz. The plate is made of a sequence of $N_{L}$ orthotropic layers. The generic *k*-th ply has a thickness $h_{k}=z_{k+1}-z_{k}$, with $z_{k+1},z_{k}$ being its upper and lower thickness coordinates. The overall plate thickness is given by $h=\sum_{k=1}^{N_{L}}h_{k}$. These geometric features are all shown and specified in Figure 1.

The three-dimensional displacements collected in $U\left(x,y,z\right)={\left\{\begin{array}{ccc} U & V & W \end{array}\right\}}^{T}$ can be written in terms of the five degrees of freedom, which are three translations u,v,w and two rotations $\phi_{x},\phi_{y}$. The vector $u\left(x,y\right)={\left\{\begin{array}{ccccc} u & v & w & \phi_{x} & \phi_{y} \end{array}\right\}}^{T}$ is conveniently introduced. The displacement field assumes the following compact aspect:

(1) $$U=I^{\left(0\right)}u+zI^{\left(1\right)}u-c_{1}F\left(I^{\left(1\right)}u+D^{\left(0\right)}I^{\left(3\right)}u\right),$$

where $D^{\left(0\right)}$ is a differential operator given by

(2) $$D^{\left(0\right)}=\left[\begin{array}{ccc} 0 & 0 & \frac{\partial }{\partial x}\\ 0 & 0 & \frac{\partial }{\partial y}\\ 0 & 0 & 0 \end{array}\right],$$

whereas the matrices $I^{\left(0\right)},I^{\left(1\right)},I^{\left(3\right)}$ are defined as

(3) $$I^{\left(0\right)}=\left[\begin{array}{ccccc} 1 & 0 & 0 & 0 & 0\\ 0 & 1 & 0 & 0 & 0\\ 0 & 0 & 1 & 0 & 0 \end{array}\right],\;I^{\left(1\right)}=\left[\begin{array}{ccccc} 0 & 0 & 0 & 1 & 0\\ 0 & 0 & 0 & 0 & 1\\ 0 & 0 & 0 & 0 & 0 \end{array}\right],\;I^{\left(3\right)}=\left[\begin{array}{ccccc} 0 & 0 & 1 & 0 & 0\\ 0 & 0 & 1 & 0 & 0\\ 0 & 0 & 1 & 0 & 0 \end{array}\right].$$

The choice of the structural theory defines the values of the constant $c_{1}$ and the thickness function $F\left(z\right)$. In particular, the TSDT is obtained for $c_{1}=\frac{4}{3h^{2}}$ and $F=z^{3}$. By setting $c_{1}=0$, the FSDT is achieved instead. On the other hand, the CLPT can be defined by using $c_{1}=1$ and F=z. The fundamental assumptions of each theory, therefore, are different according to this choice [1]. It should be specified that only the FSDT requires the shear correction factor in the definitions of the shear stresses. The value of 5/6 is considered to this aim.

The membrane strain components $\varepsilon ={\left\{\begin{array}{ccc} \varepsilon_{xx} & \varepsilon_{xy} & \gamma_{xy} \end{array}\right\}}^{T}$ and the transverse shear strains $\gamma ={\left\{\begin{array}{cc} \gamma_{xz} & \gamma_{yz} \end{array}\right\}}^{T}$ can be written as follows:

(4) $$\begin{array}{cc} \; & \varepsilon =\varepsilon^{\left(0\right)}+z\varepsilon^{\left(1\right)}+F\varepsilon^{\left(3\right)},\\ \; & \gamma =\gamma^{\left(0\right)}+F^{\prime }\gamma^{\left(2\right)}, \end{array}$$

where $F^{\prime }=\frac{\partial F}{\partial z}$. The terms introduced in (4) are discussed below. According to the notation employed by Reddy [1], the membrane strains $\varepsilon^{\left(0\right)}$ assume the following aspect:

(5) $$\varepsilon^{\left(0\right)}={\left\{\begin{array}{ccc} \varepsilon_{xx}^{\left(0\right)} & \varepsilon_{yy}^{\left(0\right)} & \gamma_{xy}^{\left(0\right)} \end{array}\right\}}^{T}=D^{\left(m\right)}I^{\left(0\right)}u.$$

On the other hand, the curvatures $\varepsilon^{\left(1\right)}$ are computed as follows:

(6) $$\varepsilon^{\left(1\right)}={\left\{\begin{array}{ccc} \varepsilon_{xx}^{\left(1\right)} & \varepsilon_{yy}^{\left(1\right)} & \gamma_{xy}^{\left(1\right)} \end{array}\right\}}^{T}=D^{\left(m\right)}I^{\left(1\right)}u,$$

where the differential operator $D^{\left(m\right)}$ is given by

(7) $$D^{\left(m\right)}=\left[\begin{array}{ccc} \frac{\partial }{\partial x} & 0 & 0\\ 0 & \frac{\partial }{\partial y} & 0\\ \frac{\partial }{\partial y} & \frac{\partial }{\partial x} & 0 \end{array}\right].$$

Higher-order membrane strains vector $\varepsilon^{\left(3\right)}$ is defined as

(8) $$\varepsilon^{\left(3\right)}={\left\{\begin{array}{ccc} \varepsilon_{xx}^{\left(3\right)} & \varepsilon_{yy}^{\left(3\right)} & \gamma_{xy}^{\left(3\right)} \end{array}\right\}}^{T}=-c_{1}\left(D^{\left(m\right)}I^{\left(1\right)}+D^{\left(b\right)}I^{\left(3\right)}\right)u,$$

in which the following definition is used for the differential operator $D^{\left(b\right)}$:

(9) $$D^{\left(b\right)}=\left[\begin{array}{ccc} 0 & 0 & \frac{\partial^{2}}{\partial x^{2}}\\ 0 & 0 & \frac{\partial^{2}}{\partial y^{2}}\\ 0 & 0 & 2\frac{\partial^{2}}{\partial x\partial y} \end{array}\right].$$

The shear strains are now discussed. The components collected in $\gamma^{\left(0\right)}$ can be written as

(10) $$\gamma^{\left(0\right)}={\left\{\begin{array}{cc} \gamma_{xz}^{\left(0\right)} & \gamma_{yz}^{\left(0\right)} \end{array}\right\}}^{T}=I_{s}^{\left(1\right)}u+D_{s}^{\left(0\right)}I_{s}^{\left(3\right)}u,$$

where

(11) $$I_{s}^{\left(1\right)}=\left[\begin{array}{ccccc} 0 & 0 & 0 & 1 & 0\\ 0 & 0 & 0 & 0 & 1 \end{array}\right],\;I_{s}^{\left(3\right)}=\left[\begin{array}{ccccc} 0 & 0 & 1 & 0 & 0\\ 0 & 0 & 1 & 0 & 0 \end{array}\right],$$

whereas the differential operator $D_{s}^{\left(0\right)}$ is given by

(12) $$D_{s}^{\left(0\right)}=\left[\begin{array}{cc} 0 & \frac{\partial }{\partial x}\\ 0 & \frac{\partial }{\partial y} \end{array}\right].$$

Likewise, higher-order shear terms included in $\gamma^{\left(2\right)}$ are defined as

(13) $$\gamma^{\left(2\right)}={\left\{\begin{array}{cc} \gamma_{xz}^{\left(2\right)} & \gamma_{yz}^{\left(2\right)} \end{array}\right\}}^{T}=-c_{1}\left(I_{s}^{\left(1\right)}u+D_{s}^{\left(0\right)}I_{s}^{\left(3\right)}u\right).$$

The governing equations are derived by means of the principle of virtual displacements [1]

(14) δΦ+δL=0,

in which δΦ is the strain energy variation, whereas δL represents the work done by applied external forces. If a laminated composite plate made of $N_{L}$ layers is considered, the variation δΦ can be defined as

(15) $$\delta \Phi =\sum_{k=1}^{N_{L}}\int_{A}\int_{z_{k}}^{z_{k+1}}\left(\delta \varepsilon^{T}\sigma^{\left(k\right)}+\delta \gamma^{T}\tau^{\left(k\right)}\right)\;dzdA,$$

in which A denotes the plate middle surface. The membrane stresses in the *k*-th layer are specified by $\sigma^{\left(k\right)}={\left\{\begin{array}{ccc} \sigma_{xx}^{\left(k\right)} & \sigma_{yy}^{\left(k\right)} & \sigma_{xy}^{\left(k\right)} \end{array}\right\}}^{T}$ and assume the following aspect [77,78]:

(16) $$\sigma^{\left(k\right)}=\left(1-\ell^{2}\nabla^{2}\right){\bar{Q}}^{\left(k\right)}\varepsilon ,$$

where *ℓ* is the nonlocal parameter linked to the influence of the micro/macroscale interactions, $\nabla^{2}=\frac{\partial^{2}}{\partial x^{2}}+\frac{\partial^{2}}{\partial y^{2}}$ is the Laplacian, and ${\bar{Q}}^{\left(k\right)}$ is the plane-stress-reduced stiffness coefficients matrix given by

(17) $${\bar{Q}}^{\left(k\right)}={\left[\begin{array}{ccc} {\bar{Q}}_{11} & {\bar{Q}}_{12} & {\bar{Q}}_{16}\\ {\bar{Q}}_{12} & {\bar{Q}}_{22} & {\bar{Q}}_{26}\\ {\bar{Q}}_{16} & {\bar{Q}}_{26} & {\bar{Q}}_{66} \end{array}\right]}^{\left(k\right)}.$$

Its terms are computed as a function of the Young’s moduli $E_{1},E_{2}$; Poisson’s ratio $\nu_{12}$; and shear modulus $G_{12}$ of the orthotropic medium [1]. The constitutive law (16) takes into account the strain gradient effect. Analogously, a similar relation can be written for the shear stresses $\tau^{\left(k\right)}={\left\{\begin{array}{cc} \tau_{xz}^{\left(k\right)} & \tau_{yz}^{\left(k\right)} \end{array}\right\}}^{T}$

(18) $$\tau^{\left(k\right)}=\left(1-\ell^{2}\nabla^{2}\right){\bar{Q}}_{s}^{\left(k\right)}\gamma ,$$

in which ${\bar{Q}}_{s}^{\left(k\right)}$ is the stiffness coefficients matrix related to the shear shown below

(19) $${\bar{Q}}_{s}^{\left(k\right)}={\left[\begin{array}{cc} {\bar{Q}}_{44} & {\bar{Q}}_{45}\\ {\bar{Q}}_{45} & {\bar{Q}}_{55} \end{array}\right]}^{\left(k\right)},$$

whose terms are computed as a function of the shear moduli $G_{13},G_{23}$ [1]. It is important to highlight that both membrane and shear stresses are characterized by a classical and a nonlocal part, which is the one multiplied by *ℓ*.

The external loads can be collected into the vector $q={\left\{\begin{array}{ccccc} q_{x} & q_{y} & q_{z} & M_{x} & M_{y} \end{array}\right\}}^{T}$, which includes five load components. Consequently, the work done by external forces δL can be written as

(20) $$\begin{array}{cc} \delta L & =-\int_{A}\delta u^{T}q\;dA. \end{array}$$

The governing equations can be obtained by conveniently introducing the stress resultants as the integrals of the stress components along the thickness of the layer. The following quantities are defined:

(21) $$\begin{array}{cc} \; & N={\left\{\begin{array}{ccc} N_{xx} & N_{yy} & N_{xy} \end{array}\right\}}^{T}=\sum_{k=1}^{N_{L}}\int_{z_{k}}^{z_{k+1}}\sigma^{\left(k\right)}dz,\\ \; & M={\left\{\begin{array}{ccc} M_{xx} & M_{yy} & M_{xy} \end{array}\right\}}^{T}=\sum_{k=1}^{N_{L}}\int_{z_{k}}^{z_{k+1}}\sigma^{\left(k\right)}z\;dz,\\ \; & P={\left\{\begin{array}{ccc} P_{xx} & P_{yy} & P_{xy} \end{array}\right\}}^{T}=\sum_{k=1}^{N_{L}}\int_{z_{k}}^{z_{k+1}}\sigma^{\left(k\right)}F\;dz,\\ \; & Q={\left\{\begin{array}{cc} Q_{x} & Q_{y} \end{array}\right\}}^{T}=\sum_{k=1}^{N_{L}}\int_{z_{k}}^{z_{k+1}}\tau^{\left(k\right)}\;dz,\\ \; & R={\left\{\begin{array}{cc} R_{x} & R_{y} \end{array}\right\}}^{T}=\sum_{k=1}^{N_{L}}\int_{z_{k}}^{z_{k+1}}\tau^{\left(k\right)}F^{\prime }\;dz. \end{array}$$

The system of five differential equations in terms of stress resultants that governs the static behavior of the plates is carried out by performing the proper manipulations starting from the principle of virtual displacements [77]. By using a compact matrix form, it becomes

(22) $$\begin{array}{cc} \; & I^{(0)T}D^{(m)T}N+I^{(1)T}D^{(m)T}M^{*}+c_{1}I^{(3)T}D^{(b)T}P\\ & \;\;\;\;\;\;\;\;\;-I_{s}^{(1)T}Q^{*}+I_{s}^{(3)T}D_{s}^{(0)T}Q^{*}+q=0, \end{array}$$

where

(23) $$M^{*}=M-c_{1}P,\;Q^{*}=Q-c_{1}R.$$

Boundary conditions in terms of primary and secondary variables are obtained as shown in the book by Reddy [1], since they are not affected by the strain gradient effect.

It is convenient at this point to express the stress resultants in the fundamental Equation (22) as a function of the displacement vector u, recalling the constitutive laws (16) and (18), as well as the definitions of the strains shown in (4). The following relations are carried out, as far as the membrane stress resultants are concerned:

(24) $$\begin{array}{cc} N & =\{AD^{\left(m\right)}I^{\left(0\right)}+BD^{\left(m\right)}I^{\left(1\right)}-c_{1}E\left(D^{\left(m\right)}I^{\left(1\right)}+D^{\left(b\right)}I^{\left(3\right)}\right)\\ \; & \;-\ell^{2}[AD_{xx}^{\left(m\right)}I^{\left(0\right)}+AD_{yy}^{\left(m\right)}I^{\left(0\right)}+BD_{xx}^{\left(m\right)}I^{\left(1\right)}+BD_{yy}^{\left(m\right)}I^{\left(1\right)}\\ \; & \;\;\;\;-c_{1}E\left(D_{xx}^{\left(m\right)}I^{\left(1\right)}+D_{yy}^{\left(m\right)}I^{\left(1\right)}+D_{xx}^{\left(b\right)}I^{\left(3\right)}+D_{yy}^{\left(b\right)}I^{\left(3\right)}\right)]\}u, \end{array}$$

(25) $$\begin{array}{cc} M & =\{BD^{\left(m\right)}I^{\left(0\right)}+DD^{\left(m\right)}I^{\left(1\right)}-c_{1}F\left(D^{\left(m\right)}I^{\left(1\right)}+D^{\left(b\right)}I^{\left(3\right)}\right)\\ \; & \;-\ell^{2}[BD_{xx}^{\left(m\right)}I^{\left(0\right)}+BD_{yy}^{\left(m\right)}I^{\left(0\right)}+DD_{xx}^{\left(m\right)}I^{\left(1\right)}+DD_{yy}^{\left(m\right)}I^{\left(1\right)}\\ \; & \;\;\;\;-c_{1}F\left(D_{xx}^{\left(m\right)}I^{\left(1\right)}+D_{yy}^{\left(m\right)}I^{\left(1\right)}+D_{xx}^{\left(b\right)}I^{\left(3\right)}+D_{yy}^{\left(b\right)}I^{\left(3\right)}\right)]\}u, \end{array}$$

(26) $$\begin{array}{cc} P & =\{ED^{\left(m\right)}I^{\left(0\right)}+FD^{\left(m\right)}I^{\left(1\right)}-c_{1}H\left(D^{\left(m\right)}I^{\left(1\right)}+D^{\left(b\right)}I^{\left(3\right)}\right)\\ \; & \;-\ell^{2}[ED_{xx}^{\left(m\right)}I^{\left(0\right)}+ED_{yy}^{\left(m\right)}I^{\left(0\right)}+FD_{xx}^{\left(m\right)}I^{\left(1\right)}+FD_{yy}^{\left(m\right)}I^{\left(1\right)}\\ \; & \;\;\;\;-c_{1}H\left(D_{xx}^{\left(m\right)}I^{\left(1\right)}+D_{yy}^{\left(m\right)}I^{\left(1\right)}+D_{xx}^{\left(b\right)}I^{\left(3\right)}+D_{yy}^{\left(b\right)}I^{\left(3\right)}\right)]\}u, \end{array}$$

where the terms into the constitutive matrices A,B,D,E,F,H are given by

(27) $${\left(A,B,D,E,F,H\right)}_{ij}=\sum_{k=1}^{N_{L}}\int_{z_{k}}^{z_{k+1}}{\bar{Q}}_{ij}^{\left(k\right)}\left(1,z,z^{2},F,zF,F^{2}\right)dz$$

for i,j=1,2,6. The following differential operators that appear due to the Laplacian are also introduced: $D_{xx}^{\left(m\right)}=P_{xy}^{\left(20\right)}D^{\left(m\right)}$, $D_{yy}^{\left(m\right)}=P_{xy}^{\left(02\right)}D^{\left(m\right)}$ and $D_{xx}^{\left(b\right)}=P_{xy}^{\left(20\right)}D^{\left(b\right)}$, $D_{yy}^{\left(b\right)}=P_{xy}^{\left(02\right)}D^{\left(b\right)}$, in which $P_{xy}^{\left(pq\right)}$ is given by

(28) $$P_{xy}^{\left(pq\right)}=\frac{\partial^{p+q}}{\partial x^{p}\partial y^{q}}.$$

On the other hand, the stress resultants related to shear forces are defined as follows:

(29) $$\begin{array}{cc} Q & =\{A_{s}I_{s}^{\left(1\right)}+A_{s}D_{s}^{\left(0\right)}I_{s}^{\left(3\right)}-c_{1}L_{s}\left(I_{s}^{\left(1\right)}+D_{s}^{\left(0\right)}I_{s}^{\left(3\right)}\right)\\ \; & \;-\ell^{2}[A_{s}P_{xy}^{\left(20\right)}I_{s}^{\left(1\right)}+A_{s}P_{xy}^{\left(02\right)}I_{s}^{\left(1\right)}+A_{s}D_{sxx}^{\left(0\right)}I_{s}^{\left(3\right)}+A_{s}D_{syy}^{\left(0\right)}I_{s}^{\left(3\right)}\\ \; & \;\;\;\;-c_{1}L_{s}\left(P_{xy}^{\left(20\right)}I_{s}^{\left(1\right)}+P_{xy}^{\left(02\right)}I_{s}^{\left(1\right)}+D_{sxx}^{\left(0\right)}I_{s}^{\left(3\right)}+D_{syy}^{\left(0\right)}I_{s}^{\left(3\right)}\right)]\}u, \end{array}$$

(30) $$\begin{array}{cc} R & =\{L_{s}I_{s}^{\left(1\right)}+L_{s}D_{s}^{\left(0\right)}I_{s}^{\left(3\right)}-c_{1}N_{s}\left(I_{s}^{\left(1\right)}+D_{s}^{\left(0\right)}I_{s}^{\left(3\right)}\right)\\ \; & \;-\ell^{2}[L_{s}P_{xy}^{\left(20\right)}I_{s}^{\left(1\right)}+L_{s}P_{xy}^{\left(02\right)}I_{s}^{\left(1\right)}+L_{s}D_{sxx}^{\left(0\right)}I_{s}^{\left(3\right)}+L_{s}D_{syy}^{\left(0\right)}I_{s}^{\left(3\right)}\\ \; & \;\;\;\;-c_{1}N_{s}\left(P_{xy}^{\left(20\right)}I_{s}^{\left(1\right)}+P_{xy}^{\left(02\right)}I_{s}^{\left(1\right)}+D_{sxx}^{\left(0\right)}I_{s}^{\left(3\right)}+D_{syy}^{\left(0\right)}I_{s}^{\left(3\right)}\right)]\}u, \end{array}$$

in which the terms collected in the constitutive matrices $A_{s},L_{s},N_{s}$, for i,j=4,5, are given by

(31) $${\left(A_{s},L_{s},N_{s}\right)}_{ij}=\sum_{k=1}^{N_{L}}\int_{z_{k}}^{z_{k+1}}\kappa_{s}{\left(Q_{s}\right)}_{ij}^{\left(k\right)}\left(1,F^{\prime },{\left(F^{\prime }\right)}^{2}\right)dz,$$

where $\kappa_{s}$ is the shear correction factor. Its value is different from the unity only for the FSDT, in which it is assumed equal to 5/6. The differential operators $D_{sxx}^{\left(0\right)},D_{syy}^{\left(0\right)}$ are computed as $D_{sxx}^{\left(0\right)}=P_{xy}^{\left(20\right)}D_{s}^{\left(0\right)},D_{syy}^{\left(0\right)}=P_{xy}^{\left(02\right)}D_{s}^{\left(0\right)}$.

## 3. Solution Procedure

Once the nonlocal governing equations are written in terms of the displacements collected in u, they can be solved analytically by means of the Navier methodology. As illustrated in [1], this approach can be applied only for some peculiar lamination schemes, which are antisymmetric cross-ply and antisymmetric angle-ply, respectively. These two cases are analyzed separately in the following, assuming simply supported boundary conditions for both circumstances. The solution to the current static problem is provided by the algebraic linear system shown below

(32) KΔ=F,

in which $\Delta ={\left\{\begin{array}{ccccc} U_{mn} & V_{mn} & W_{mn} & X_{mn} & Y_{mn} \end{array}\right\}}^{T}$ is the vector that collects the unknown coefficients that determine the displacement amplitudes. On the other hand, K and F are the stiffness matrix and the load vector, respectively. The terms included in the symmetric matrix K will be specified for each lamination scheme. On the other hand, the load vector has the following definition, assuming that only transverse surface forces are applied: $F={\left\{\begin{array}{ccccc} 0 & 0 & Q_{mn} & 0 & 0 \end{array}\right\}}^{T}$, where $Q_{mn}$ is equal to $q_{z}$ for a sinusoidally distributed load, having introduced the same expansion also for the applied external forces [1].

### 3.1. Cross-Ply Laminates

In order to apply the Navier approach for the cross-ply sequence at issue, the stiffnesses written below are all equal to zero:

(33) $$\begin{array}{c} A_{16}=A_{26}=A_{s45}=B_{16}=B_{26}=D_{16}=D_{26}=0,\\ E_{16}=E_{26}=F_{16}=F_{26}=H_{16}=H_{26}=L_{s45}=N_{s45}=0. \end{array}$$

In this circumstance, the solution can be sought assuming the following expansion:

(34) $$u=\sum_{n=1}^{\infty }\sum_{m=1}^{\infty }\Delta \circ \left\{\begin{array}{c} \cos\alpha x\sin\beta y\\ \sin\alpha x\cos\beta y\\ \sin\alpha x\sin\beta y\\ \cos\alpha x\sin\beta y\\ \sin\alpha x\cos\beta y \end{array}\right\},$$

where ○ stands for the elementwise product and α=mπ/a,β=nπ/b. The explicit expressions of the coefficients $K_{ij}$ included in the stiffness matrix K, for i,j=1,…,5, whose size is clearly 5×5 are listed in Appendix A, recalling that $K_{ij}=K_{ji}$.

### 3.2. Angle-Ply Laminates

The Navier approach can be applied for angle-ply laminates if the following stiffnesses are all equal to zero:

(35) $$\begin{array}{c} A_{16}=A_{26}=A_{s45}=B_{11}=B_{12}=B_{22}=B_{66}=D_{16}=D_{26}=0,\\ E_{11}=E_{12}=E_{22}=E_{66}=F_{16}=F_{26}=H_{16}=H_{26}=L_{s45}=N_{s45}=0. \end{array}$$

In this case, the solution is obtained assuming the following expansion:

(36) $$u=\sum_{n=1}^{\infty }\sum_{m=1}^{\infty }\Delta \circ \left\{\begin{array}{c} \sin\alpha x\cos\beta y\\ \cos\alpha x\sin\beta y\\ \sin\alpha x\sin\beta y\\ \cos\alpha x\sin\beta y\\ \sin\alpha x\cos\beta y \end{array}\right\}.$$

The expressions of the coefficients $K_{ij}$, for i,j=1,…,5, of the stiffness matrix K, are listed in Appendix A, presenting only those terms that are different from the ones valid for cross-ply sequences.

The solution in terms of Δ can be easily obtained from Equation (32), for both laminates under consideration. Once the amplitudes in Δ are computed, definitions (34) and (36) allow us to obtain the displacements within the reference domain. Consequently, the strains can be also deduced. The membrane stress components $\sigma^{\left(k\right)}$ can be evaluated as well, following the procedure illustrated in the book by Reddy [1] through the constitutive relations previously presented. On the other hand, the shear stress components $\tau^{\left(k\right)}$ are determined from the three-dimensional equilibrium elasticity equations [1,73]. The complete procedure is omitted for conciseness purposes.

## 4. Numerical Results

The current Section aims to present the results of the static analyses. Due to the general features of the theoretical approach, the solutions are presented for different nonlocal theories, which are CLPT, FSDT and TSDT, setting properly the values of $c_{1}$ and F. As far as the mechanical features are concerned, the ratio between the longitudinal and transverse Young’s moduli $E_{1}/E_{2}$ is specified in each application, whereas the other quantities are taken as $G_{12}=G_{13}=0.5E_{2}$, $G_{23}=0.2E_{2}$, $\nu_{12}=0.25$ for Material 1, or $G_{12}=G_{13}=0.6E_{2}$, $G_{23}=0.5E_{2}$, $\nu_{12}=0.25$ for Material 2. The lamination schemes, instead, are denoted by $\left(\theta^{\left(1\right)}/\ldots /\theta^{\left(k\right)}/\ldots /\theta^{\left(N_{L}\right)}\right)$, where $\theta^{\left(k\right)}$ stands for the orientation of the *k*-th layer. The results are presented in dimensionless form. In particular, the central deflection $\bar{w}$ is given by

(37) $$\bar{w}=w\left(\frac{a}{2},\frac{b}{2}\right)\frac{E_{2}h^{3}}{a^{4}q_{z}}.$$

On the other hand, the stress components are evaluated as follows, unless differently specified:

(38) $$\begin{array}{c} {\bar{\sigma }}_{xx}=\sigma_{xx}\left(\frac{a}{2},\frac{b}{2},\frac{h}{2}\right)\frac{h^{2}}{b^{2}q_{z}},\;{\bar{\sigma }}_{yy}=\sigma_{yy}\left(\frac{a}{2},\frac{b}{2},\frac{h}{4}\right)\frac{h^{2}}{b^{2}q_{z}},\;{\bar{\sigma }}_{xy}=\sigma_{xy}\left(0,0,\frac{h}{2}\right)\frac{h^{2}}{b^{2}q_{z}},\\ {\bar{\sigma }}_{xz}=\sigma_{xz}\left(0,\frac{b}{2},0\right)\frac{h}{bq_{z}},\;{\bar{\sigma }}_{yz}=\sigma_{yz}\left(\frac{a}{2},0,0\right)\frac{h}{bq_{z}}. \end{array}$$

It should be specified that the values of the stresses presented in this Section are all related to the classical component, which can be deducted from definitions (16) and (18) following the approach used in [73,77]. The analyses are carried out for increasing values of the dimensionless nonlocal parameter ${\left(\ell /a\right)}^{2}$, in order to show the effect of the strain gradient on the static solutions.

**Figure 2:**
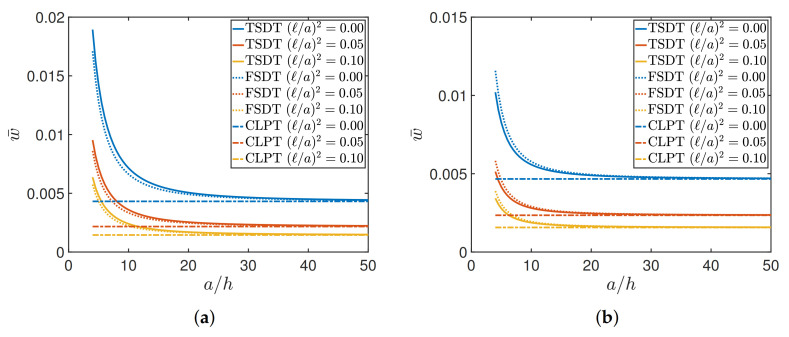
Dimensionless central deflection $\bar{w}$ versus side-to-thickness ratio a/h of a square plate subjected to a sinusoidally distributed load varying the nonlocal ratio ${\left(\ell /a\right)}^{2}$, for different lamination schemes: (**a**) cross-ply (0/90/90/0); (**b**) angle-ply (45/−45). TSDT—Third-order Shear Deformation Theory; FSDT—First-order Shear Deformation Theory; CLPT—Classical Laminated Plate Theory.

The first application aims to investigate the central deflection $\bar{w}$ as a function of side-to-thickness ratio a/h of a square plate for different lamination schemes: cross-ply (0/90/90/0) and angle-ply (45/−45). The results are shown in Figure 2, assuming $E_{1}/E_{2}=25$ for the cross-ply (Material 1) and $E_{1}/E_{2}=40$ for the angle-ply (Material 2).

The graphs include the structural response obtained by means of the three theories. Each model is related to a different line style: solid line for TSDT, dotted for FSDT, and dash-dotted for CLPT. The color, instead, is linked to the value of the nonlocal parameter. The same choice is also kept in the following figures. It can be observed that the greater is the nonlocal effect and the lower is the vertical deflection, independently from the theory. In other words, the central deflection is reduced by increasing the value of the nonlocal parameter ${\left(\ell /a\right)}^{2}$. The FSDT and TSDT, moreover, are characterized by a comparable behavior and are highly affected by plate thickness. By increasing the ratio a/h, their displacements tend to the results of the CLPT, which do not depend on that geometric ratio. The corresponding curves, in fact, are described by rectilinear functions. Similar behaviors are obtained in both lamination schemes.

In the next test, a (0/90/0) cross-ply square plate is considered. Material 1 is taken into account in this circumstance, assuming $E_{1}/E_{2}=25$. The results are presented in Table 1 for different values of a/h, varying the structural theory. Where available, the analytical solutions by Reddy [1] are provided, in terms of both displacement and stress components. The reference results are clearly presented only for classical elasticity, assuming ${\left(\ell /a\right)}^{2}=0$. The comparison proves a very good agreement between the current approach and the reference one.

Table 2, instead, aims to extend these results to the nonlocal elasticity framework, varying the ratio ${\left(\ell /a\right)}^{2}$. The same reduction of the displacement values observed in Figure 2 can also be seen numerically in this circumstance. The three theories provide really close results for thin plates even if the strain gradient effect is taken into account. It should be noted from these tables that noticeable differences can be seen especially in terms of membrane stresses in thick configurations (defined by a/h=4), if the results related to the different theories are compared. As it will be highlighted in the following paragraphs, this is due to the fact that the TSDT is characterized by nonlinear stress profiles. This nonlinearity is particularly emphasized for thicker plates, whereas it is reduced for lower values of a/h.

A (0/90/90/0) cross-ply lamination scheme is considered in the next application. Even in this case, Material 1 is taken into account to describe the orthotropic features of the layers, with $E_{1}/E_{2}=25$. The results are shown in Table 3 for the classical elasticity. In the same Table, the solutions shown in [1] are presented for comparison purposes.

As in the previous test, the present configuration is analyzed also in the nonlocal framework. Results for increasing values of ${\left(\ell /a\right)}^{2}$ are included in Table 4 for the sake of completeness.

The reference solutions are all related to classical elasticity. In the following application, the current methodology is also verified with regard to nonlocal elasticity, taking into account the solutions presented in [77]. To this aim, only CLPT is considered in accordance with the reference paper, due to the availability of the literature. Several cross-ply lamination schemes are considered, assuming $E_{1}/E_{2}=25$ and Material 1 as orthotropic features. The results are presented in Table 5 for various stacking sequences, varying the value of ${\left(\ell /a\right)}^{2}$, in terms of central deflections and membrane stress components. In this circumstance, the following dimensionless quantities are considered for the stresses:

(39) $$\begin{array}{c} {\bar{\sigma }}_{yy}=\sigma_{yy}\left(\frac{a}{2},\frac{b}{2},\frac{h}{2}\right)\frac{h^{2}}{b^{2}q_{z}},\;{\bar{\sigma }}_{xy}=\sigma_{xy}\left(a,b,-\frac{h}{2}\right)\frac{h^{2}}{b^{2}q_{z}}, \end{array}$$

whereas the same expression introduced before is used for ${\bar{\sigma }}_{xx}$. The results shown in Table 5 prove that the present solutions are in very good agreement with the reference ones, also in the framework of nonlocal elasticity.

The next application is focused on the bending analysis of antisymmetric angle ply laminates, characterized by (θ/−θ/…) as the lamination scheme and θ being the arbitrary orientation of each layer. Firstly, the analyses are presented in terms of dimensionless central deflection $\bar{w}$, considering square plates made of Material 2 and $E_{1}/E_{2}=40$ as orthotropic ratio. The results are presented for several side-to-thickness values a/h, orthotropic angles θ, and different structural theories in Table 6, as far as classical elasticity is concerned, which means ${\left(\ell /a\right)}^{2}=0$. The values are in good agreement with the ones taken as references [1].

As in the previous cases, the analyses are extended to nonlocal elasticity by increasing the value of ${\left(\ell /a\right)}^{2}$ but keeping the same geometric ratios and mechanical features. The results are presented in Table 7. Even in these circumstances, the differences between TSDT and FSDT decrease for lower values of thickness, and the displacements tend to the ones of the CLPT.

A ${\left(-45/45\right)}_{4}$ laminated plate square plate is considered in the next application. Each orthotropic layer is made of Material 1, with $E_{1}/E_{2}=25$. This test aims to evaluate the stress components in angle-ply configurations. With respect to the dimensionless values shown in (38), only the transverse shear stress ${\bar{\sigma }}_{xz}$ is specified in a different thickness coordinate as specified below

(40) $$\begin{array}{c} {\bar{\sigma }}_{xz}=\sigma_{xz}\left(0,\frac{b}{2},\frac{h}{2}\right)\frac{h}{bq_{z}}. \end{array}$$

It should be recalled that in the following application ${\bar{\sigma }}_{xx}={\bar{\sigma }}_{yy}$ and ${\bar{\sigma }}_{xz}={\bar{\sigma }}_{yz}$, therefore, their values are not repeated twice. The results related to the classical elasticity are shown in Table 8, varying a/h and the structural theory. Even if the displacements become closer independently from the considered approach, the stresses assume different values especially if the thickness is greater. In this circumstance, the reference solutions are available only for the FSDT [1].

On the other hand, the nonlocal counterpart of the current application is shown in Table 9. The increasing values of ${\left(\ell /a\right)}^{2}$ emphasize the differences in terms of stresses, especially if the plates are thicker.

**Figure 3:**
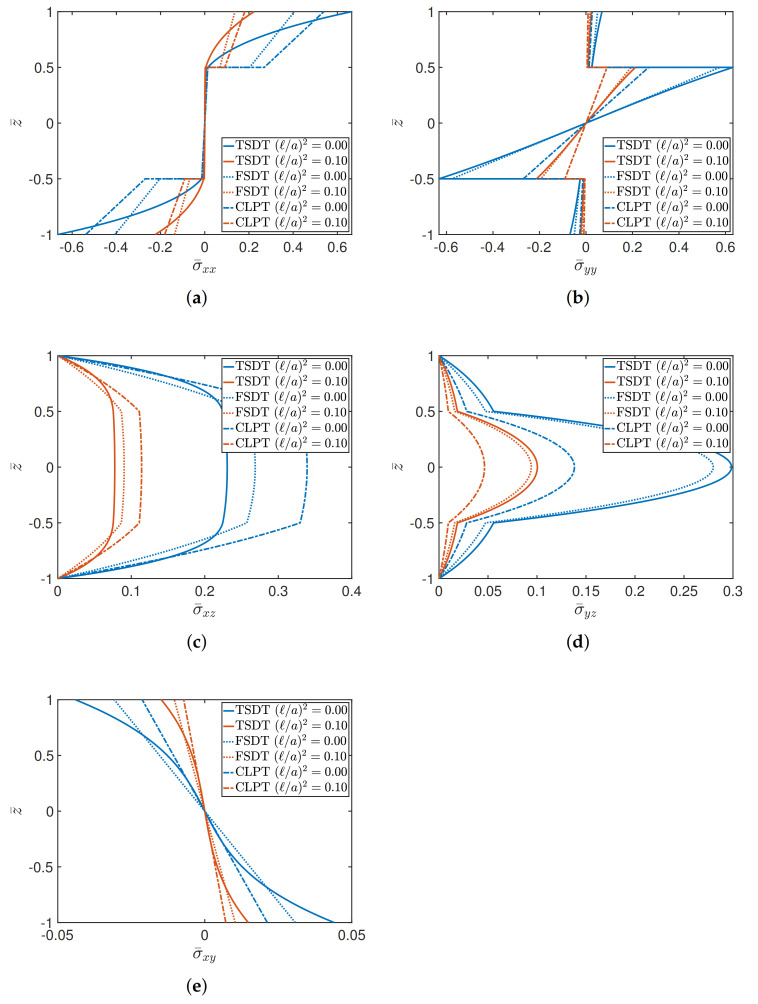
Through-the-thickness distributions of stresses of a (0/90/90/0) square plate characterized by a/h=4 for different theories and values of ${\left(\ell /a\right)}^{2}$. The following stress components are considered: (**a**) ${\bar{\sigma }}_{xx}$; (**b**) ${\bar{\sigma }}_{yy}$; (**c**) ${\bar{\sigma }}_{xz}$; (**d**) ${\bar{\sigma }}_{yz}$; (**e**) ${\bar{\sigma }}_{xy}$.

**Figure 4:**
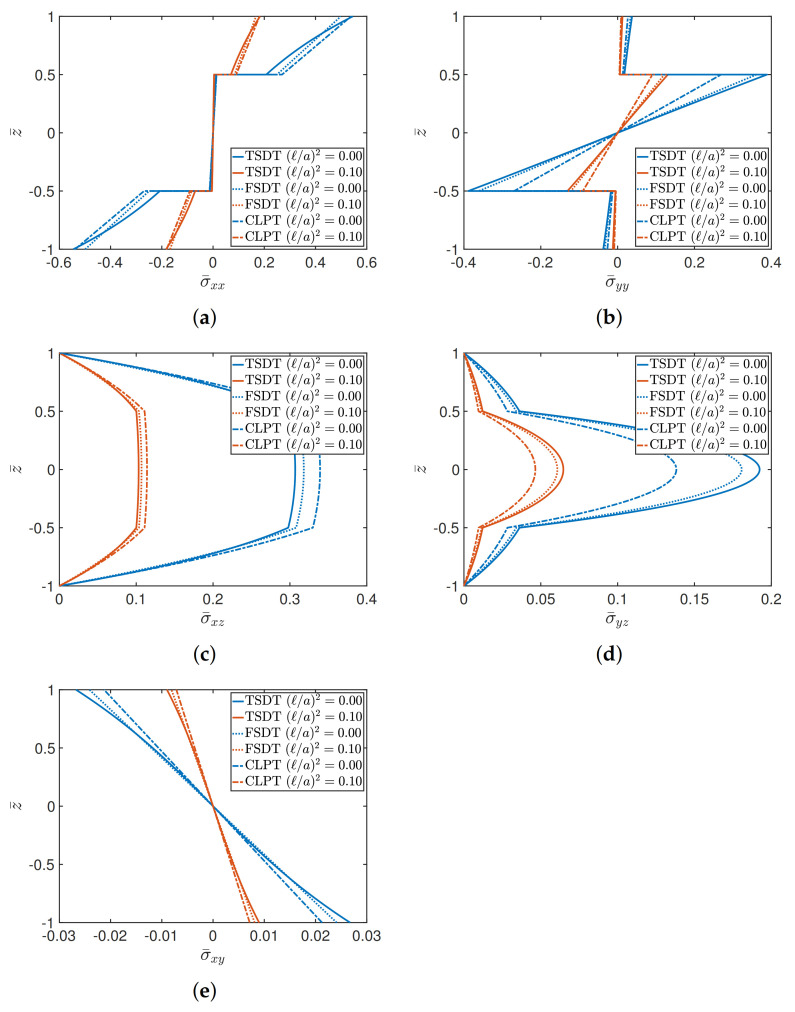
Through-the-thickness distributions of stresses of a (0/90/90/0) square plate characterized by a/h=10, for different theories and values of ${\left(\ell /a\right)}^{2}$. The following stress components are considered: (**a**) ${\bar{\sigma }}_{xx}$; (**b**) ${\bar{\sigma }}_{yy}$; (**c**) ${\bar{\sigma }}_{xz}$; (**d**) ${\bar{\sigma }}_{yz}$; (**e**) ${\bar{\sigma }}_{xy}$.

The last tests aim to present the stress analysis in terms of the through-the-thickness distributions of the various components, highlighting the differences that could arise by varying the structural approach (TSDT, FSDT, and CLPT) for different geometric ratios. The effect of ${\left(\ell /a\right)}^{2}$ is also investigated. Firstly, a (0/90/90/0) cross-ply square plate is analyzed, for a/h equal to 4 and 10, respectively. The orthotropic features of the layers are obtained by setting $E_{1}/E_{2}=25$ and selecting Material 1 as constituent. The membrane and shear dimensionless stresses are given by

(41) $$\begin{array}{c} {\bar{\sigma }}_{xx}=\sigma_{xx}\left(\frac{a}{2},\frac{b}{2},\bar{z}\right)\frac{h^{2}}{b^{2}q_{z}},\;{\bar{\sigma }}_{yy}=\sigma_{yy}\left(\frac{a}{2},\frac{b}{2},\bar{z}\right)\frac{h^{2}}{b^{2}q_{z}},\;{\bar{\sigma }}_{xy}=\sigma_{xy}\left(0,0,\bar{z}\right)\frac{h^{2}}{b^{2}q_{z}},\\ {\bar{\sigma }}_{xz}=\sigma_{xz}\left(0,\frac{b}{2},\bar{z}\right)\frac{h}{bq_{z}},\;{\bar{\sigma }}_{yz}=\sigma_{yz}\left(\frac{a}{2},0,\bar{z}\right)\frac{h}{bq_{z}}, \end{array}$$

where $\bar{z}=2z/h$ stands for the dimensionless thickness coordinate. The through-the-thickness stress distributions in Figure 3 are related to the case a/h=4, which is representative for thick plates. It can be observed that in both classical and nonlocal elasticity, the TSDT is characterized by significantly different profiles due to the higher-order features of the displacement field. This aspect is more evident in the membrane stress components, which are obtained by means of the application of constitutive laws. In fact, it should be recalled that the TSDT allows a cubic representation of the stress profiles, whereas a linear variation is associated with the FSDT and CLPT. This feature gives rise to noticeable differences if thick plates are investigated, as it can be seen from the plot of ${\bar{\sigma }}_{xx}$ in Figure 3. The variation of the transverse stresses, instead, is always characterized by curved and continuous profiles since they are equilibrium-derived, according to the procedure shown in [1]. A lower value of thickness is considered in the graphs shown in Figure 4, assuming a/h=10. Due to this choice, the stress distributions tend to the same value, for both ${\left(\ell /a\right)}^{2}=0$ and ${\left(\ell /a\right)}^{2}=0.10$, independently from the theory. Therefore, the differences among the theories is practically negligible starting from a/h=10, which is typically the geometric ratio that characterizes moderately thick plates.

A similar analysis is carried out for a (−45/45) angle-ply square plate, for a/h equal to 4 and 10, respectively. In this case, the orthotropic features of the layers are given by $E_{1}/E_{2}=40$. Material 2 is taken into account as constituent. With respect to the dimensionless expressions introduced in (41), a different value of ${\bar{\sigma }}_{yz}$ is considered, which is defined below:

(42) $$\begin{array}{c} {\bar{\sigma }}_{yz}=\sigma_{yz}\left(0,\frac{b}{2},\bar{z}\right)\frac{h}{bq_{z}}. \end{array}$$

**Figure 5:**
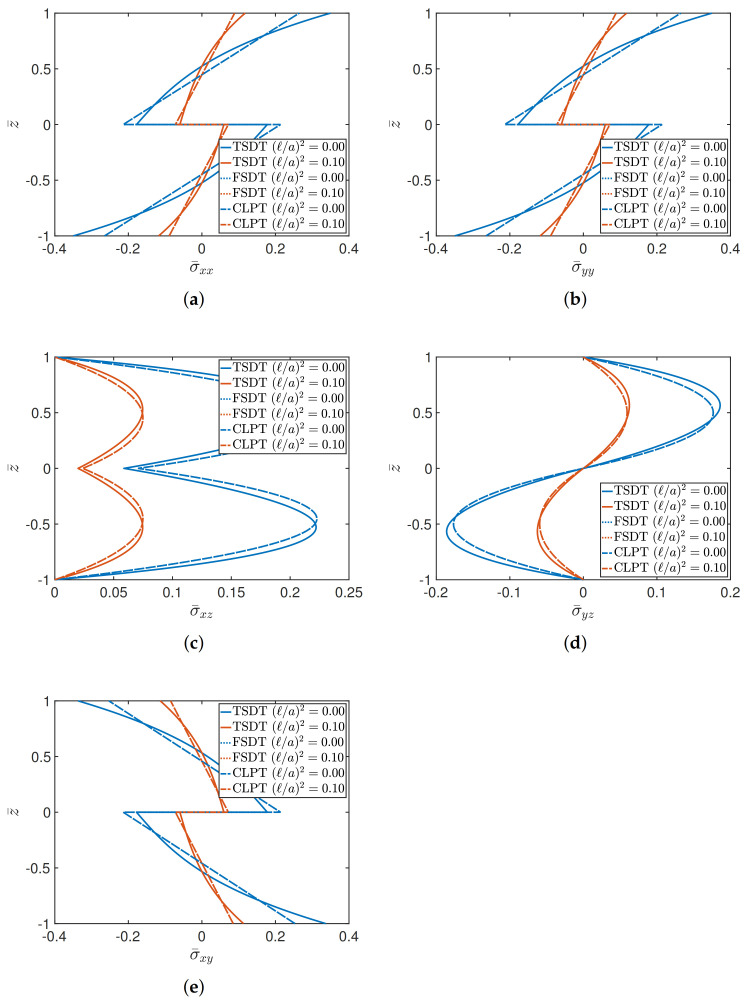
Through-the-thickness distributions of stresses of a (−45/45) square plate characterized by a/h=4 for different theories and values of ${\left(\ell /a\right)}^{2}$. The following stress components are considered: (**a**) ${\bar{\sigma }}_{xx}$; (**b**) ${\bar{\sigma }}_{yy}$; (**c**) ${\bar{\sigma }}_{xz}$; (**d**) ${\bar{\sigma }}_{yz}$; (**e**) ${\bar{\sigma }}_{xy}$.

**Figure 6:**
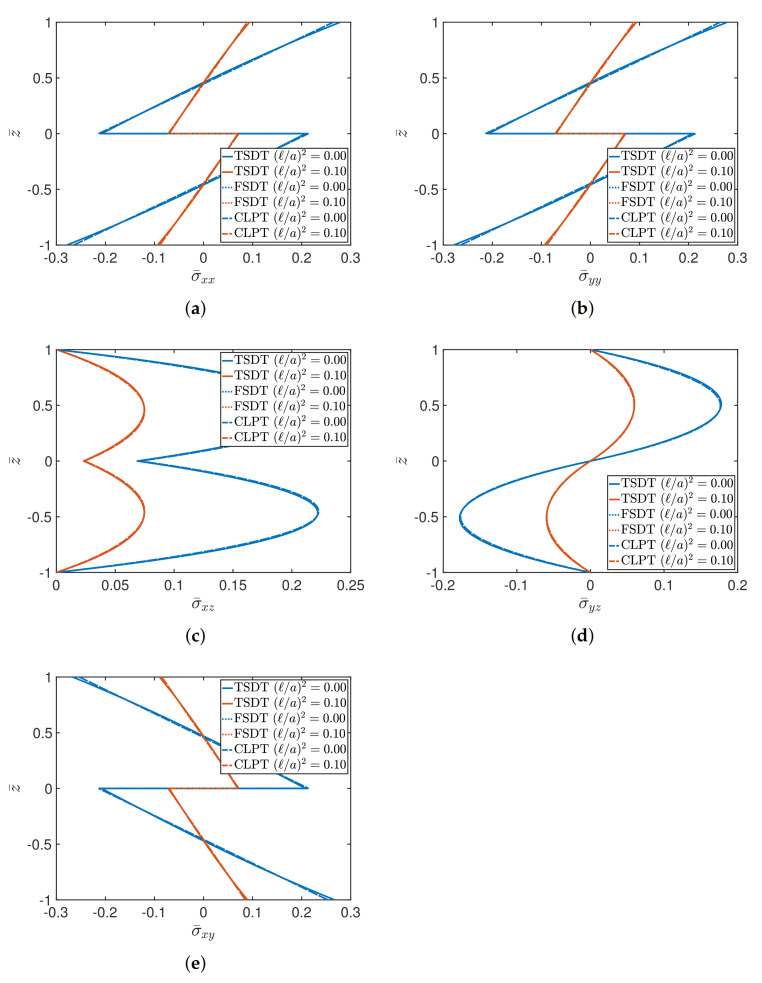
Through-the-thickness distributions of stresses of a (−45/45) square plate characterized by a/h=10, for different theories and values of ${\left(\ell /a\right)}^{2}$. The following stress components are considered: (**a**) ${\bar{\sigma }}_{xx}$; (**b**) ${\bar{\sigma }}_{yy}$; (**c**) ${\bar{\sigma }}_{xz}$; (**d**) ${\bar{\sigma }}_{yz}$; (**e**) ${\bar{\sigma }}_{xy}$.

The graphical plots are shown in Figure 5 and Figure 6 for a/h=4 and a/h=10, respectively. It can be observed that in these circumstances, the FSDT and CLPT are overlapped. The nonlinear behavior of TSDT is clearly more evident for thicker plates, for both classical and nonlocal elasticity.

## 5. Conclusions

A theoretical framework able to simultaneously and accurately study thick and thin laminated composite plates has been presented in the paper. In particular, the chosen kinematic model is able to describe several theories if properly set, which are the CLPT, FSDT, and TSDT. The theories have been modified to include the strain gradient effect, in order to take into account nonlocal contributions in the evaluation of stresses. The proposed approach is general, and the fundamental static equations have been written for arbitrary configurations by using a compact matrix notation. In order to provide an analytical solution, the Navier methodology has been applied to deal with cross-ply and angle-ply lamination schemes. The explicit definitions needed in the solution procedure have been presented, highlighting also the contributions linked to the strain gradient effect. The approach has been validated numerically by means of comparison with the results accessible in the literature, if available, for both classical and nonlocal elasticity. The results have been presented in terms of displacements and stresses, varying the geometric features, mechanical properties, lamination schemes, as well as the influence of the nonlocal effect. The differences that have been observed among the theories have been emphasized. In particular, the results have proven that the stress components are noticeably different by changing the plate theory, especially for higher values of thickness. Finally, it should be specified that the proposed solutions could be used for further advancements of this topic in nonlocal elasticity and could be taken as benchmarks for future comparisons, especially if numerical methods are developed.

## Figures and Tables

**Table 1:** Dimensionless central displacement and stress components of a cross-ply square plate with (0/90/0) as the lamination scheme, for ${\left(\ell /a\right)}^{2}=0$.

a/h	Theory	$10^{2}\bar{w}$	${\bar{\sigma }}_{\mathrm{xx}}$	${\bar{\sigma }}_{\mathrm{yy}}$	${\bar{\sigma }}_{\mathrm{yz}}$	${\bar{\sigma }}_{\mathrm{xz}}$	${\bar{\sigma }}_{\mathrm{xy}}$
4	TSDT [1]	1.9218	0.7345	-	0.2086	-	-
	TSDT	1.9217	0.7344	0.0315	0.2086	0.2855	0.0497
	FSDT [1]	1.7758	0.4370	-	0.1968	-	-
	FSDT	1.7757	0.4370	0.0307	0.1968	0.3368	0.0369
10	TSDT [1]	0.7125	0.5684	-	0.1167	-	-
	TSDT	0.7125	0.5684	0.0183	0.1167	0.3693	0.0277
	FSDT [1]	0.6693	0.5134	-	0.1108	-	-
	FSDT	0.6693	0.5134	0.0176	0.1108	0.3806	0.0252
100	TSDT [1]	0.4342	0.5390	-	0.0827	-	-
	TSDT	0.4342	0.5390	0.0134	0.0827	0.3948	0.0214
	FSDT [1]	0.4337	0.5384	-	0.0827	-	-
	FSDT	0.4337	0.5384	0.0134	0.0827	0.3950	0.0213
	CLPT [1]	0.4313	0.5387	-	0.0823	-	-
	CLPT	0.4312	0.5387	0.0133	0.0823	0.3951	0.0213

**Table 2:** Dimensionless central displacement and stress components of a cross-ply square plate with (0/90/0) as the lamination scheme, varying the nonlocal ratio ${\left(\ell /a\right)}^{2}$.

${\left(\ell /a\right)}^{2}$	a/h	Theory	$10^{2}\bar{w}$	${\bar{\sigma }}_{\mathrm{xx}}$	${\bar{\sigma }}_{\mathrm{yy}}$	${\bar{\sigma }}_{\mathrm{yz}}$	${\bar{\sigma }}_{\mathrm{xz}}$	${\bar{\sigma }}_{\mathrm{xy}}$
0.05	4	TSDT	0.9672	0.3696	0.0158	0.1050	0.1437	0.0250
		FSDT	0.8937	0.2199	0.0154	0.0991	0.1695	0.0186
	10	TSDT	0.3586	0.2860	0.0092	0.0587	0.1859	0.0139
		FSDT	0.3368	0.2584	0.0089	0.0558	0.1916	0.0127
	100	TSDT	0.2185	0.2713	0.0067	0.0416	0.1987	0.0107
		FSDT	0.2183	0.2710	0.0067	0.0416	0.1988	0.0107
		CLPT	0.2170	0.2711	0.0067	0.0414	0.1989	0.0107
0.1	4	TSDT	0.6462	0.2470	0.0106	0.0701	0.0960	0.0167
		FSDT	0.5971	0.1469	0.0103	0.0662	0.1132	0.0124
	10	TSDT	0.2396	0.1911	0.0062	0.0392	0.1242	0.0093
		FSDT	0.2251	0.1726	0.0059	0.0373	0.1280	0.0085
	100	TSDT	0.1460	0.1812	0.0045	0.0278	0.1328	0.0072
		FSDT	0.1458	0.1810	0.0045	0.0278	0.1328	0.0072
		CLPT	0.1450	0.1811	0.0045	0.0277	0.1329	0.0072

**Table 3:** Dimensionless central displacement and stress components of a cross-ply square plate with (0/90/90/0) as the lamination scheme, for ${\left(\ell /a\right)}^{2}=0$.

a/h	Theory	$10^{2}\bar{w}$	${\bar{\sigma }}_{\mathrm{xx}}$	${\bar{\sigma }}_{\mathrm{yy}}$	${\bar{\sigma }}_{\mathrm{yz}}$	${\bar{\sigma }}_{\mathrm{xz}}$	${\bar{\sigma }}_{\mathrm{xy}}$
4	TSDT [1]	1.8940	0.6650	0.6320	0.2390	0.2060	0.0440
	TSDT	1.8936	0.6651	0.6322	0.2985	0.2305	0.0440
	FSDT [1]	1.7100	0.4060	0.5760	0.1960	0.1400	0.0308
	FSDT	1.7095	0.4059	0.5764	0.2799	0.2686	0.0308
10	TSDT [1]	0.7150	0.5460	0.3890	0.1530	0.2640	0.0268
	TSDT	0.7147	0.5456	0.3888	0.1924	0.3069	0.0268
	FSDT [1]	0.6630	0.4989	0.3610	0.1300	0.1670	0.0241
	FSDT	0.6627	0.4989	0.3614	0.1807	0.3181	0.0241
20	TSDT [1]	0.5060	0.5390	0.3040	0.1230	0.2820	0.0228
	TSDT	0.5060	0.5393	0.3043	0.1541	0.3299	0.0228
	FSDT [1]	0.4910	0.5270	0.2960	0.1090	0.1750	0.0221
	FSDT	0.4912	0.5273	0.2956	0.1503	0.3332	0.0221
100	TSDT [1]	0.4340	0.5390	0.2710	0.1120	0.2900	0.0213
	TSDT	0.4343	0.5387	0.2708	0.1389	0.3389	0.0213
	FSDT [1]	0.4340	0.5380	0.2700	0.1010	0.1780	0.0213
	FSDT	0.4337	0.5382	0.2704	0.1387	0.3390	0.0213
	CLPT [1]	0.4310	0.5390	0.2690	0.1380	0.3390	0.0213
	CLPT	0.4312	0.5387	0.2693	0.1382	0.3393	0.0213

**Table 4:** Dimensionless central displacement and stress components of a cross-ply square plate with (0/90/90/0) as the lamination scheme, varying the nonlocal ratio ${\left(\ell /a\right)}^{2}$.

${\left(\ell /a\right)}^{2}$	a/h	Theory	$10^{2}\bar{w}$	${\bar{\sigma }}_{\mathrm{xx}}$	${\bar{\sigma }}_{\mathrm{yy}}$	${\bar{\sigma }}_{\mathrm{yz}}$	${\bar{\sigma }}_{\mathrm{xz}}$	${\bar{\sigma }}_{\mathrm{xy}}$
0.05	4	TSDT	0.9530	0.3347	0.3182	0.1502	0.1160	0.0222
		FSDT	0.8604	0.2043	0.2901	0.1409	0.1352	0.0155
	10	TSDT	0.3597	0.2746	0.1957	0.0968	0.1545	0.0135
		FSDT	0.3335	0.2511	0.1819	0.0909	0.1601	0.0121
	20	TSDT	0.2547	0.2714	0.1531	0.0776	0.1661	0.0115
		FSDT	0.2472	0.2654	0.1488	0.0757	0.1677	0.0111
	100	TSDT	0.2186	0.2711	0.1363	0.0699	0.1705	0.0107
		FSDT	0.2183	0.2709	0.1361	0.0698	0.1706	0.0107
		CLPT	0.2170	0.2711	0.1356	0.0696	0.1707	0.0107
0.1	4	TSDT	0.6367	0.2236	0.2126	0.1004	0.0775	0.0148
		FSDT	0.5748	0.1365	0.1938	0.0941	0.0903	0.0104
	10	TSDT	0.2403	0.1835	0.1307	0.0647	0.1032	0.0090
		FSDT	0.2228	0.1678	0.1215	0.0608	0.1070	0.0081
	20	TSDT	0.1702	0.1813	0.1023	0.0518	0.1109	0.0077
		FSDT	0.1652	0.1773	0.0994	0.0506	0.1120	0.0074
	100	TSDT	0.1460	0.1811	0.0911	0.0467	0.1139	0.0072
		FSDT	0.1458	0.1810	0.0909	0.0466	0.1140	0.0072
		CLPT	0.1450	0.1811	0.0906	0.0465	0.1141	0.0072

**Table 5:** Dimensionless central displacement and membrane stress components of several cross-ply square plates, varying the nonlocal ratio ${\left(\ell /a\right)}^{2}$. The reference solutions (Ref.) are taken from [77].

		$10^{2}\bar{w}$		${\bar{\sigma }}_{\mathrm{xx}}$		${\bar{\sigma }}_{\mathrm{yy}}$		${\bar{\sigma }}_{\mathrm{xy}}$	
${\left(\ell /a\right)}^{2}$	Scheme	Ref. [77]	Present	Ref. [77]	Present	Ref. [77]	Present	Ref. [77]	Present
0.05	(0)	0.2170	0.2170	0.2711	0.2711	0.0134	0.0134	0.0107	0.0107
	(0/90)	0.5353	0.5352	0.0424	0.0424	0.3602	0.3602	0.0264	0.0264
	${\left(0/90\right)}_{2}$	0.2549	0.2549	0.0180	0.0180	0.2450	0.2450	0.0126	0.0126
	${\left(0/90\right)}_{4}$	0.2254	0.2254	0.0149	0.0149	0.2491	0.2491	0.0111	0.0111
0.1	(0)	0.1450	0.1450	0.1811	0.1811	0.0090	0.0090	0.0072	0.0072
	(0/90)	0.3576	0.3576	0.0284	0.0284	0.2407	0.2406	0.0176	0.0176
	${\left(0/90\right)}_{2}$	0.1703	0.1703	0.0120	0.0120	0.1637	0.1637	0.0084	0.0084
	${\left(0/90\right)}_{4}$	0.1506	0.1506	0.0100	0.0100	0.1664	0.1664	0.0074	0.0074

**Table 6:** Dimensionless central displacement $10^{2}\bar{w}$ of a angle-ply square plate with (θ/−θ/…) as lamination scheme, for ${\left(\ell /a\right)}^{2}=0$. The number of plies is denoted by $N_{L}$.

		$\theta =5^{{}^{\circ}}$		$\theta =30^{{}^{\circ}}$		$\theta =45^{{}^{\circ}}$	
a/h	Theory	$N_{L}=2$	$N_{L}=6$	$N_{L}=2$	$N_{L}=6$	$N_{L}=2$	$N_{L}=6$
4	TSDT [1]	1.2625	1.2282	1.0838	0.8851	1.0203	0.8375
	TSDT	1.2624	1.2282	1.0836	0.8851	1.0202	0.8375
	FSDT [1]	1.3165	1.2647	1.2155	0.8994	1.1576	0.8531
	FSDT	1.3165	1.2647	1.2154	0.8994	1.1575	0.8531
10	TSDT [1]	0.4848	0.4485	0.5916	0.3007	0.5581	0.2745
	TSDT	0.4848	0.4485	0.5915	0.3006	0.5580	0.2745
	FSDT [1]	0.4883	0.4491	0.6099	0.2989	0.5773	0.2728
	FSDT	0.4882	0.4491	0.6098	0.2989	0.5772	0.2728
20	TSDT [1]	0.3579	0.3209	0.5180	0.2127	0.4897	0.1905
	TSDT	0.3579	0.3209	0.5179	0.2127	0.4896	0.1905
	FSDT [1]	0.3586	0.3208	0.5224	0.2121	0.4944	0.1899
	FSDT	0.3585	0.3208	0.5224	0.2121	0.4943	0.1899
100	TSDT [1]	0.3162	0.2789	0.4942	0.1842	0.4676	0.1634
	TSDT	0.3162	0.2789	0.4941	0.1842	0.4676	0.1634
	FSDT [1]	0.3162	0.2789	0.4944	0.1842	0.4678	0.1633
	FSDT	0.3162	0.2789	0.4943	0.1842	0.4678	0.1633
	CLPT [1]	0.3145	0.2771	0.4932	0.1831	0.4667	0.1622
	CLPT	0.3144	0.2771	0.4931	0.1831	0.4667	0.1622

**Table 7:** Dimensionless central displacement $10^{2}\bar{w}$ of a angle-ply square plate with (θ/−θ/…) as lamination scheme, varying the nonlocal ratio ${\left(\ell /a\right)}^{2}$. The number of plies is denoted by $N_{L}$.

			$\theta =5^{{}^{\circ}}$		$\theta =30^{{}^{\circ}}$		$\theta =45^{{}^{\circ}}$	
${\left(\ell /a\right)}^{2}$	a/h	Theory	$N_{L}=2$	$N_{L}=6$	$N_{L}=2$	$N_{L}=6$	$N_{L}=2$	$N_{L}=6$
0.05	4	TSDT	0.6353	0.6181	0.5454	0.4455	0.5134	0.4215
		FSDT	0.6625	0.6365	0.6117	0.4527	0.5825	0.4293
	10	TSDT	0.2440	0.2257	0.2977	0.1513	0.2808	0.1382
		FSDT	0.2457	0.2260	0.3069	0.1504	0.2905	0.1373
	20	TSDT	0.1801	0.1615	0.2606	0.1070	0.2464	0.0959
		FSDT	0.1804	0.1614	0.2629	0.1067	0.2488	0.0956
	100	TSDT	0.1591	0.1404	0.2487	0.0927	0.2353	0.0822
		FSDT	0.1591	0.1403	0.2488	0.0927	0.2354	0.0822
		CLPT	0.1583	0.1395	0.2482	0.0921	0.2349	0.0816
0.1	4	TSDT	0.4245	0.4130	0.3644	0.2976	0.3431	0.2816
		FSDT	0.4427	0.4253	0.4087	0.3024	0.3892	0.2868
	10	TSDT	0.1630	0.1508	0.1989	0.1011	0.1876	0.0923
		FSDT	0.1642	0.1510	0.2051	0.1005	0.1941	0.0917
	20	TSDT	0.1203	0.1079	0.1741	0.0715	0.1646	0.0640
		FSDT	0.1206	0.1079	0.1756	0.0713	0.1662	0.0638
	100	TSDT	0.1063	0.0938	0.1662	0.0620	0.1572	0.0549
		FSDT	0.1063	0.0938	0.1662	0.0619	0.1573	0.0549
		CLPT	0.1057	0.0932	0.1658	0.0616	0.1569	0.0546

**Table 8:** Dimensionless central displacement and stress components of a cross-ply square plate with ${\left(-45/45\right)}_{4}$ as lamination scheme, for ${\left(\ell /a\right)}^{2}=0$.

a/h	Theory	$10^{2}\bar{w}$	${\bar{\sigma }}_{\mathrm{xx}}$	${\bar{\sigma }}_{\mathrm{xy}}$	${\bar{\sigma }}_{\mathrm{xz}}$
4	TSDT	1.2931	0.2511	0.2406	0.2116
	FSDT	1.3317	0.1445	0.1384	0.2487
10	TSDT	0.4207	0.1623	0.1554	0.2425
	FSDT [1]	0.4198	0.1445	0.1384	0.2487
	FSDT	0.4198	0.1445	0.1384	0.2487
20	TSDT	0.2900	0.1490	0.1427	0.2471
	FSDT [1]	0.2896	0.1445	0.1384	0.2487
	FSDT	0.2896	0.1445	0.1384	0.2487
100	TSDT	0.2479	0.1447	0.1386	0.2486
	FSDT [1]	0.2479	0.1445	0.1384	0.2487
	FSDT	0.2479	0.1445	0.1384	0.2487
	CLPT [1]	0.2462	0.1445	0.1384	0.2487
	CLPT	0.2461	0.1445	0.1384	0.2487

**Table 9:** Dimensionless central displacement and stress components of a cross-ply square plate with ${\left(-45/45\right)}_{4}$ as lamination scheme, varying the nonlocal ratio ${\left(\ell /a\right)}^{2}$.

${\left(\ell /a\right)}^{2}$	a/h	Theory	$10^{2}\bar{w}$	${\bar{\sigma }}_{\mathrm{xx}}$	${\bar{\sigma }}_{\mathrm{xy}}$	${\bar{\sigma }}_{\mathrm{xz}}$
0.05	4	TSDT	0.6508	0.1264	0.1211	0.1065
		FSDT	0.6702	0.0727	0.0697	0.1251
	10	TSDT	0.2117	0.0817	0.0782	0.1220
		FSDT	0.2113	0.0727	0.0697	0.1251
	20	TSDT	0.1460	0.0750	0.0718	0.1244
		FSDT	0.1457	0.0727	0.0697	0.1251
	100	TSDT	0.1248	0.0728	0.0697	0.1251
		FSDT	0.1248	0.0727	0.0697	0.1251
		CLPT	0.1239	0.0727	0.0697	0.1251
0.1	4	TSDT	0.4348	0.0844	0.0809	0.0711
		FSDT	0.4478	0.0486	0.0465	0.0836
	10	TSDT	0.1415	0.0546	0.0523	0.0815
		FSDT	0.1412	0.0486	0.0465	0.0836
	20	TSDT	0.0975	0.0501	0.0480	0.0831
		FSDT	0.0974	0.0486	0.0465	0.0836
	100	TSDT	0.0834	0.0486	0.0466	0.0836
		FSDT	0.0834	0.0486	0.0465	0.0836
		CLPT	0.0828	0.0486	0.0465	0.0836

## Data Availability

Data sharing not applicable.

## Appendix A. Algebraic Form of the Stiffness Matrix

As specified in (32), the static problems at issue are governed by the algebraic linear system KΔ=F. The terms included in the symmetric matrix K are presented in this Appendix, for the two cases investigated in the paper.

### Appendix A.1. Cross-Ply Laminates

As far as cross-ply lamination schemes are concerned, the terms of the first row of the matrix K are given by

(A1) $$\begin{array}{cc} K_{11} & =A_{11}\alpha^{2}+A_{66}\beta^{2}+\ell^{2}[A_{11}(\alpha^{4}+\alpha^{2}\beta^{2})+A_{66}\left(\beta^{4}+\alpha^{2}\beta^{2}\right)], \end{array}$$

(A2) $$\begin{array}{cc} K_{12} & =\left(A_{12}+A_{66}\right)\alpha \beta +\ell^{2}[\left(A_{12}+A_{66}\right)(\alpha^{3}\beta +\alpha \beta^{3})], \end{array}$$

(A3) $$\begin{array}{cc} K_{13} & =-c_{1}\{E_{11}\alpha^{3}+\left(E_{12}+2E_{66}\right)\alpha \beta^{2}\\ \; & \;\;+\ell^{2}[E_{11}(\alpha^{5}+\alpha^{3}\beta^{2})+\left(E_{12}+2E_{66}\right)(\alpha^{3}\beta^{2}+\alpha \beta^{4})]\}, \end{array}$$

(A4) $$\begin{array}{cc} K_{14} & =\left(B_{11}-c_{1}E_{11}\right)\alpha^{2}+\left(B_{66}-c_{1}E_{66}\right)\beta^{2}\\ \; & \;\;+\ell^{2}[\left(B_{11}-c_{1}E_{11}\right)(\alpha^{4}+\alpha^{2}\beta^{2})+\left(B_{66}-c_{1}E_{66}\right)(\beta^{4}+\alpha^{2}\beta^{2})\}, \end{array}$$

(A5) $$\begin{array}{cc} K_{15} & =\left(B_{12}-c_{1}E_{12}+B_{66}-c_{1}E_{66}\right)\alpha \beta \\ \; & \;\;+\ell^{2}[\left(B_{12}-c_{1}E_{12}+B_{66}-c_{1}E_{66}\right)(\alpha^{3}\beta +\alpha \beta^{3})]. \end{array}$$

The terms of the second row are defined as

(A6) $$\begin{array}{cc} K_{22} & =A_{66}\alpha^{2}+A_{22}\beta^{2}+\ell^{2}[A_{66}(\alpha^{4}+\alpha^{2}\beta^{2})+A_{22}(\beta^{4}+\alpha^{2}\beta^{2})], \end{array}$$

(A7) $$\begin{array}{cc} K_{23} & =-c_{1}\{E_{22}\beta^{3}+\left(E_{12}+2E_{66}\right)\alpha^{2}\beta \\ \; & \;\;+\ell^{2}[E_{22}(\alpha \beta^{3}+\beta^{5})+\left(E_{12}+2E_{66}\right)(\alpha^{4}\beta +\alpha^{2}\beta^{3})]\}, \end{array}$$

(A8) $$\begin{array}{cc} K_{24} & =\left(B_{12}-c_{1}E_{12}+B_{66}-c_{1}E_{66}\right)\alpha \beta \\ \; & \;\;+\ell^{2}[\left(B_{12}-c_{1}E_{12}+B_{66}-c_{1}E_{66}\right)(\alpha^{3}\beta +\alpha \beta^{3})], \end{array}$$

(A9) $$\begin{array}{cc} K_{25} & =\left(B_{22}-c_{1}E_{22}\right)\beta^{2}+\left(B_{66}-c_{1}E_{66}\right)\alpha^{2}\\ \; & \;\;+\ell^{2}[\left(B_{22}-c_{1}E_{22}\right)(\alpha^{2}\beta^{2}+\beta^{4})+\left(B_{66}-c_{1}E_{66}\right)(\alpha^{4}+\alpha^{2}\beta^{2})]. \end{array}$$

As far as the terms of the third row are concerned, one gets

(A10) $$\begin{array}{cc} K_{33} & =(A_{s44}-2c_{1}L_{s44}+c_{1}^{2}N_{s44})\alpha^{2}+(A_{s55}-2c_{1}L_{s55}+c_{1}^{2}N_{s55})\beta^{2}\\ \; & \;\;+\ell^{2}[(A_{s44}-2c_{1}L_{s44}+c_{1}^{2}N_{s44})(\alpha^{4}+\alpha^{2}\beta^{2})\\ \; & \;\;+(A_{s55}-2c_{1}L_{s55}+c_{1}^{2}N_{s55})(\beta^{4}+\alpha^{2}\beta^{2})]\\ \; & \;\;+c_{1}^{2}\{H_{11}\alpha^{4}+2\left(H_{12}+2H_{66}\right)\alpha^{2}\beta^{2}+H_{22}\beta^{4}\\ \; & \;\;+\ell^{2}[H_{11}(\alpha^{6}+\alpha^{4}\beta^{2})+2\left(H_{12}+2H_{66}\right)(\alpha^{4}\beta^{2}+\alpha^{2}\beta^{4})\\ \; & \;\;+H_{22}(\beta^{6}+\alpha^{2}\beta^{4})]\}, \end{array}$$

(A11) $$\begin{array}{cc} K_{34} & =(A_{s44}-2c_{1}L_{s44}+c_{1}^{2}N_{s44})\alpha \\ \; & \;\;+\ell^{2}[(A_{s44}-2c_{1}L_{s44}+c_{1}^{2}N_{s44})(\alpha^{3}+\alpha \beta^{2})]\\ \; & \;\;-c_{1}\{\left(F_{11}-c_{1}H_{11}\right)\alpha^{3}+\left(F_{12}-c_{1}H_{12}+2F_{66}-2c_{1}H_{66}\right)\alpha \beta^{2}\\ \; & \;\;+\ell^{2}[\left(F_{11}-c_{1}H_{11}\right)(\alpha^{5}+\alpha^{3}\beta^{2})\\ & \;\;+\left(F_{12}-c_{1}H_{12}+2F_{66}-2c_{1}H_{66}\right)(\alpha^{3}\beta^{2}+\alpha \beta^{4})]\}, \end{array}$$

(A12) $$\begin{array}{cc} K_{35} & =(A_{s55}-2c_{1}L_{s55}+c_{1}^{2}N_{s55})\alpha \\ \; & \;\;+\ell^{2}[(A_{s55}-2c_{1}L_{s55}+c_{1}^{2}N_{s55})(\alpha^{3}+\alpha \beta^{2})]\\ \; & \;\;-c_{1}\{\left(F_{22}-c_{1}H_{22}\right)\beta^{3}+\left(F_{12}-c_{1}H_{12}+2F_{66}-2c_{1}H_{66}\right)\alpha^{2}\beta \\ \; & \;\;+\ell^{2}[\left(F_{22}-c_{1}H_{22}\right)(\beta^{5}+\alpha^{2}\beta^{3})\\ & \;\;+\left(F_{12}-c_{1}H_{12}+2F_{66}-2c_{1}H_{66}\right)(\alpha^{4}\beta +\alpha^{2}\beta^{3})]\}. \end{array}$$

Instead, the terms of the fourth row are given by

(A13) $$\begin{array}{cc} K_{44} & =A_{s44}-2c_{1}L_{s44}+c_{1}^{2}N_{s44}\\ \; & \;\;+\ell^{2}[(A_{s44}-2c_{1}L_{s44}+c_{1}^{2}N_{s44})(\alpha^{2}+\beta^{2})]\\ \; & \;\;+(D_{11}-2c_{1}F_{11}+c_{1}^{2}H_{11})\alpha^{2}+(D_{66}-2c_{1}F_{66}+c_{1}^{2}H_{66})\beta^{2}\\ \; & \;\;+(D_{11}-2c_{1}F_{11}+c_{1}^{2}H_{11})(\alpha^{4}+\alpha^{2}\beta^{2})\\ \; & \;\;+(D_{66}-2c_{1}F_{66}+c_{1}^{2}H_{66})(\beta^{4}+\alpha^{2}\beta^{2})]\}, \end{array}$$

(A14) $$\begin{array}{cc} K_{45} & =(D_{12}-2c_{1}F_{12}+c_{1}^{2}H_{12})\alpha \beta +(D_{66}-2c_{1}F_{66}+c_{1}^{2}H_{66})\alpha \beta \\ \; & \;\;+\ell^{2}[(D_{12}-2c_{1}F_{12}+c_{1}^{2}H_{12})(\alpha^{3}\beta +\alpha \beta^{3})\\ \; & \;\;+(D_{66}-2c_{1}F_{66}+c_{1}^{2}H_{66})(\alpha^{3}\beta +\alpha \beta^{3})]\}. \end{array}$$

Finally, the terms included in the last row assume the following aspect:

(A15) $$\begin{array}{cc} K_{55} & =A_{s55}-2c_{1}L_{s55}+c_{1}^{2}N_{s55}\\ \; & \;\;+\ell^{2}[(A_{s55}-2c_{1}L_{s55}+c_{1}^{2}N_{s55})(\alpha^{2}+\beta^{2})]\\ \; & \;\;+(D_{66}-2c_{1}F_{66}+c_{1}^{2}H_{66})\alpha^{2}+(D_{22}-2c_{1}F_{22}+c_{1}^{2}H_{22})\beta^{2}\\ \; & \;\;+(D_{66}-2c_{1}F_{66}+c_{1}^{2}H_{66})(\alpha^{4}+\alpha^{2}\beta^{2})\\ \; & \;\;+(D_{22}-2c_{1}F_{22}+c_{1}^{2}H_{22})(\beta^{4}+\alpha^{2}\beta^{2})]\}. \end{array}$$

### Appendix A.2. Angle-Ply Laminates

The coefficients included in the stiffness matrix are presented below for angle-ply laminates. When omitted, their definitions are the same as those presented for the cross-ply sequences. The terms of the first row are given by

(A16) $$\begin{array}{cc} K_{13} & =-c_{1}\{3E_{16}\alpha^{2}\beta +E_{26}\beta^{3}+\ell^{2}[3E_{16}(\alpha^{4}\beta +\alpha^{2}\beta^{3})+E_{26}(\alpha^{2}\beta^{3}+\beta^{5})]\}, \end{array}$$

(A17) $$\begin{array}{cc} K_{14} & =2\left(B_{16}-c_{1}E_{16}\right)\alpha \beta +\ell^{2}[2\left(B_{16}-c_{1}E_{16}\right)(\alpha^{3}\beta +\alpha \beta^{3})], \end{array}$$

(A18) $$\begin{array}{cc} K_{15} & =\left(B_{16}-c_{1}E_{16}\right)\alpha^{2}+\left(B_{26}-c_{1}E_{26}\right)\beta^{2}\\ \; & \;\;+\ell^{2}[\left(B_{16}-c_{1}E_{16}\right)(\alpha^{4}+\alpha^{2}\beta^{2})+\left(B_{26}-c_{1}E_{26}\right)(\alpha^{2}\beta^{2}+\beta^{4})]. \end{array}$$

The terms of the second row are defined as

(A19) $$\begin{array}{cc} K_{23} & =-c_{1}\{E_{16}\alpha^{3}+3E_{26}\alpha \beta^{2}+\ell^{2}[E_{16}(\alpha^{5}+\alpha^{3}\beta^{2})+3E_{26}(\alpha^{3}\beta^{2}+\alpha \beta^{4})]\}, \end{array}$$

(A20) $$\begin{array}{cc} K_{24} & =\left(B_{16}-c_{1}E_{16}\right)\alpha^{2}+\left(B_{26}-c_{1}E_{26}\right)\beta^{2}\\ \; & \;\;+\ell^{2}[\left(B_{16}-c_{1}E_{16}\right)(\alpha^{4}+\alpha^{2}\beta^{2})+\left(B_{26}-c_{1}E_{26}\right)(\alpha^{2}\beta^{2}+\beta^{4})], \end{array}$$

(A21) $$\begin{array}{cc} K_{25} & =2\left(B_{26}-c_{1}E_{26}\right)\alpha \beta +\ell^{2}[2\left(B_{26}-c_{1}E_{26}\right)(\alpha^{3}\beta +\alpha \beta^{3})]. \end{array}$$
